# Anti-atherosclerotic effects of the glucagon-like peptide-1 (GLP-1) based therapies in patients with type 2 Diabetes Mellitus: A meta-analysis

**DOI:** 10.1038/srep10202

**Published:** 2015-06-26

**Authors:** Xiaoyan Song, Hetang Jia, Yuebo Jiang, Liang Wang, Yan Zhang, Yiming Mu, Yu Liu

**Affiliations:** 1 Department of Endocrinology, Chinese PLA General Hospital, Beijing 100853, China; 2 Department of Endocrinology, Chinese PLA 309 Hospital, Beijing 100091, China; 3 Department of Acupuncture, Chinese PLA General Hospital, Beijing 100853, China; 4 Department of Orthopedics, Chinese PLA 309 Hospital, Beijing 100091; 5 Department of Geriatric Endocrinology, General Hospital of PLA, Beijing 100853, China

## Abstract

This study assessed the effect of GLP-1 based therapies on atherosclerotic markers in type 2 diabetes patients. 31 studies were selected to obtain data after multiple database searches and following inclusion and exclusion criteria. Age and BMI of the participants of longitudinal studies were 59.8 ± 8.3 years and 29.2 ± 5.7 kg/m^2^ (Mean±SD). Average duration of GLP-1 based therapies was 20.5 weeks. Percent flow-mediated diameter (%FMD) did not change from baseline significantly but when compared to controls, %FMD increased non-significantly following GLP-1-based therapies (1.65 [−0.89, 4.18]; P = 0.2; REM) in longitudinal studies and increased significantly in cross sectional studies (2.58 [1.68, 3.53]; P < 0.00001). Intima media thickness decreased statistically non-significantly by the GLP-1 based therapies. GLP-1 based therapies led to statistically significant reductions in the serum levels of brain natriuretic peptide (−40.16 [−51.50, −28.81]; P < 0.0001; REM), high sensitivity c-reactive protein (−0.27 [−0.48, −0.07]; P = 0.009), plasminogen activator inhibitor-1 (−12.90 [−25.98, 0.18]; P=0.05), total cholesterol (−5.47 [−9.55, −1.39]; P = 0.009), LDL-cholesterol (−3.70 [−7.39, −0.00]; P = 0.05) and triglycerides (−16.44 [−25.64, −7.23]; P = 0.0005) when mean differences with 95% CI in the changes from baselines were meta-analyzed. In conclusion, GLP-1-based therapies appear to provide beneficial effects against atherosclerosis. More randomized data will be required to arrive at conclusive evidence.

Type 2 Diabetes Mellitus (T2D) is a progressive disease with gradually increasing prevalence. Currently, 340 million people are suffering globally and it is estimated that, by 2030, it would be the seventh leading cause of mortality^1^ Microvascular complications associated with diabetes leads to organ loss, and both stroke and heart disease related mortality is 2–4 times higher in diabetics than in normal individuals^2^.

Atherosclerosis is one of the major possible consequences of insulin resistance, obesity and T2D^3^. The vascular endothelial dysfunction is a critical step of atherogenesis which is usually characterized by endothelium-dependent vasodilatation and vasoconstriction imbalance and the disturbed balance of antithrombotic and prothrombotic factors^4^. At an earlier stage, apolipoprotein B starts retaining in the sub-endothelial spaces which activates endothelial cells to secrete chemokines which then recruit monocytes. Adhesion molecules of the endothelial cells make contacts with monocytes which later invade the vascular wall and differentiate into macrophages. The macrophages uptake lipids and lipoproteins and secrete cytokines and chemokines to further recruit CD4 T cells and smooth myocytes in the intima layer of vessels. This leads to an advance stage of atherogenesis in which plaques contain lipids, cholesterol, macrophages and altered myocytes^5^.

A category of gut peptide hormones, the incretins (glucagon-lie peptide 1; GLP-1 and glucose-dependent insulinotropic polypeptide; GIP), released during meals with increasing glucose levels in the blood are insulinotropic that are responsible for about three quarters of total insulin secretion^6^. In subjects with T2D, the GLP-1 secretion is impaired and its insulinotropic and glucagon suppressive actions are weakened. Therapeutic use of GLP-1 tends to normalize alpha and beta cell sensitivity to glucose leading to improved glycemic control and reduced diabetic toxicity^7^. Besides modulating a range of physiological effects such as blood glucose and metabolism regulation, the GLP-1 is also thought to prevent multiple steps of atherosclerosis^4^.

A number of studies have shown beneficial effects of GLP-1-based interventions on measures affecting cardiovascular pathologies such as blood pressure, body weight, and lipid metabolism that are thought to be exerted independent of GLP-1 analogue’s effects on glycemic control^8^,^9^ and the effects on markers of atherosclerosis are also reported^10^,^11^,^12^. However, data regarding the effects of GLP-1 based therapies on atherosclerosis generated in various studies is not previously reviewed systematically. Importantly, outcomes of various studies are not consistent which provides rationale for a meta-analysis of the relevant studies. The purpose of the present study is to meta-analyze data pertaining to regarding the effects of GLP-1 based therapies on markers of atherosclerosis observed in related clinical trials.

## Method

### Literature Search

PRISMA (Preferred Reporting Items for Systematic Reviews and Meta-Analyses) guidelines were followed in carrying out this study. The literature search was made in EBSCO, Embase, Medline/PubMed, Ovid, and Web of Science electronic databases. Important MeSH terms and keywords used were: glucagon-like peptide 1 (GLP-1), GLP-1 analogue, incretin, dipeptidyl peptidase 4 (DPP-4) inhibitor, atherosclerosis, arteriosclerosis, atherogenesis, plaque, intima media thickness (IMT), flow-mediated dilation (FMD), adiponectin, leptin, endothelin, E-selectin, resistin, pentraxin, brain natriuretic peptide (BNP), monocyte chemotactic protein (MCP), vascular cell adhesion molecule-1 (VCAM-1), plasminogen activator inhibitor-1 (PAI-1), tumor necrosis factor-alpha (TNF-α), interleukin-6 (IL-6), high sensitivity C-reactive protein (hs-CRP), triglyceride and cholesterol. Relevant research articles published during 1990 and September 2014 were retrieved, and corroborations and cross references of important research papers were also searched.

### Inclusion and exclusion criteria

Rationale for the inclusion of studies in the present meta-analytical review was to assess the effects of increased GLP-1 levels in blood on atheroscelortic markers in T2D patients. The inclusion criteria were: a) study investigated the effects of GLP-1-based therapies on atherosclerosis by examining the effects of either exogenous GLP-1, or GLP-1 analogue, or any other serum GLP-1 elevating agent; b) study utilized a longitudinal design and reported at least one marker of atherosclerosis with numerical values of baseline and endpoint samples or mentioned measured changes from baseline; c) study utilized a cross sectional design to study changes in % FMD following a GLP-1-based therapy. The exclusion criteria were: a) studies involving cell culture experiments to examine the effects of GLP-1-based therapies on atherosclerosis markers; and b) cross sectional studies examining the effects of GLP-1-based therapies on atherosclerosis markers other than % FMD; and c) studies with longitudinal designs providing either baseline or endpoint measures only or providing information inadequate for meta-analysis.

### Primary and secondary endpoints

Primary endpoints of the present study were changes in % FMD, IMT, PAI-1, VCAM-1, MCP-1, BNP, hs-CRP, IL-6, TNF-α, endothelin, E-selectin, and resistin while the secondary endpoints were the changes in serum levels of adiponectin, leptin, total cholesterol, HDL-cholesterol, LDL-cholesterol, and triglyceride.

### Data extraction, synthesis and statistical analysis

Study screening and selection was carried out by two authors independently with high inter-rater reliability (kappa = 0.93). The data regarding the participants’ demographic, clinical and therapeutic characteristics, outcomes and other important parameters were extracted from tabular, textual and graphic sources of the published research papers independently by two researchers and were cross-checked later (kappa = 0.95). Dis-agreements were mutually discussed and resolved but when agreement could not be reached, a third researcher was involved.

For the assessment of the changes from baseline following GLP-1-based therapies in % FMD and IMT, mean differences with 95% confidence intervals (CI) were calculated between baseline samples and end-of-treatment samples for each study and then an overall effect size was generated. For controlled trials’ data, changes from the baseline were first measured and then mean differences [95% CI] between GLP-1-based therapy patients and controls were calculated for each study and then overall effect size of the meta-analysis was generated.

Meta-analyses were performed with RevMan (Version 5.2; Cochrane Collaboration) software by using means and standard deviation values of the selected markers of atherosclerosis of GLP-1 based therapy patients / controls. The overall effect of treatment was calculated as weighted average of the inverse variance adjusted individual effects. Significance of difference between GLP-1-based therapies and controls was tested with z test. Both fixed- (FEM) and random-effect (REM) models were utilized in the meta-analyses for outcome interpretation depending on between-study heterogeneity which was tested by I^2^ index. Sensitivity analyses were performed to explore the sources of higher statistical heterogeneity and funnel plots were examined for the assessment of publication bias.

## Results

Thirty one studies^13^,^14^,^15^,^16^,^17^,^18^,^19^,^20^,^21^,^22^,^23^,^24^,^25^,^26^,^27^,^28^,^29^,^30^,^31^,^32^,^33^,^34^,^35^,^36^,^37^,^38^,^39^,^40^,^41^,^42^,^43^ were selected after following inclusion and exclusion criteria. Study screening and selection procedure is summarized in Fig. S1. Among the included studies, 24 utilized longitudinal designs (10 with placebo- and 10 comparator-controlled^13^,^14^,^15^,^16^,^17^,^18^,^19^,^20^,^21^,^22^,^23^,^24^,^25^,^26^,^27^,^28^,^29^,^30^,^31^,^32^ and 4 were single armed)^33^,^34^,^35^,^36^. Seven controlled cross sectional studies examining the acute effects of GLP-1-based therapies on FMD were also included^37^,^38^,^39^,^40^,^41^,^42^,^43^. Average age and BMI of the participants of the longitudinal studies were 59.8 ± 8.3 years and 29.2 ± 5.7 kg/m^2^. The average duration of GLP-1 based therapies was 20.5 weeks (range 4–52).

**Figure 1:**
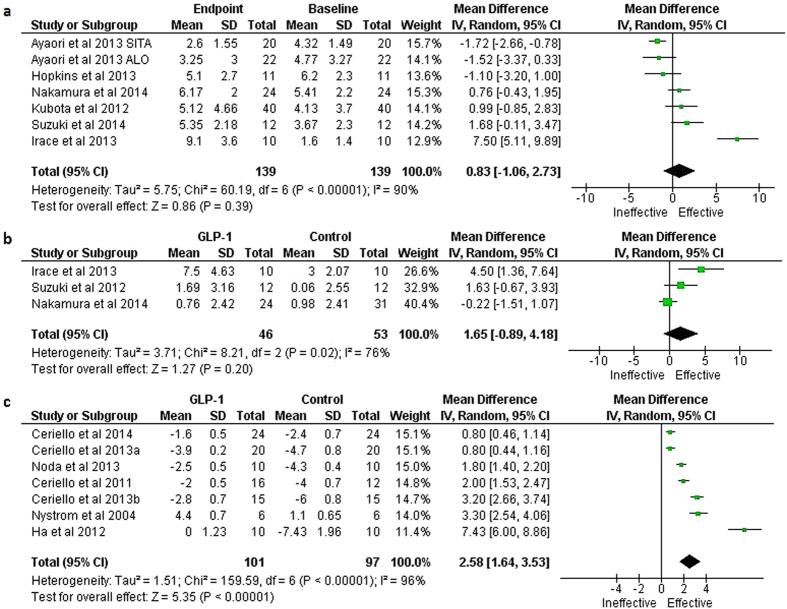
Forest plots showing the effects of GLP-1-based therapies on % FMD: **a**) meta-analysis of the changes from baseline following GLP-1 based therapies in longitudinal studies, **b** Mean difference between GLP-1 based therapies and controls in longitudinal studies, and **c**) mean difference between GLP-1 based therapy patients and controls in cross sectional studies examining acute effects.

Major findings of the meta-analyses are presented in Table 1. A meta-analysis of 6 studies^13^,^20^,^30^,^32^,^33^,^35^ which attempted to estimate overall effect size of the changes from baseline in % FMD following GLP-1-based therapies could not find any significant difference between baseline and end-of-treatment samples (mean change from baseline was 0.83 [−1.06, 2.73] %; P = 0.39; REM; Fig. 1a). In the meta-analysis of 3 studies^20^,^28^,^32^ which compared a GLP-1-based therapy against a comparator, GLP-1-based therapies were found to increase % FMD non-significantly (mean difference [95% CI] in the changes from baseline: 1.65 [−0.89, 4.18] %; P = 0.2; REM; Fig. 1b). However, 7 cross sectional studies examining acute effects of the GLP-1-based therapies^37^,^38^,^39^,^40^,^41^,^42^,^43^ found a significant increase in % FMD following GLP-1-based interventions (mean difference in the changes from baseline: 2.58 [1.68, 3.53] %; P < 0.00001; REM; Fig. 1c).

**Figure 2:**
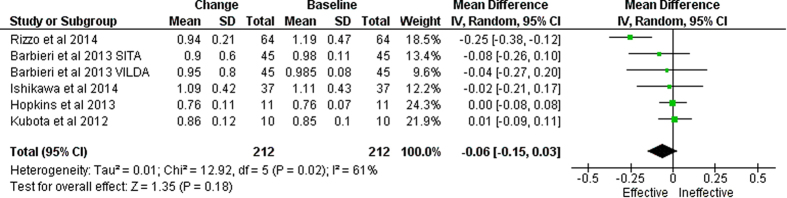
Forest plot showing meta-analysis of the changes in intima media from baseline in six studies which examined the effect of GLP-1 based therapies in longitudinal designs.

Five studies^14^,^21^,^33^,^35^,^36^, were identified which evaluated the effect of GLP-1-based therapies on IMT using longitudinal designs. A meta-analysis of these studies revealed a statistically non-significant decrease in IMT with a mean change from baseline of −0.06 [−0.15, 0.03] mm; P = 0.18; REM (Fig. 2). Two studies^14^,^21^ were identified that evaluated the effect of GLP-1-based therapies in a controlled longitudinal design. A meta-analysis of these revealed a statistically non-significant reduction in IMT (Mean difference between GLP-1-based therapies and controls −0.04 [−0.09, 0.01]; P = 0.1; FEM/REM).

**Figure 3:**
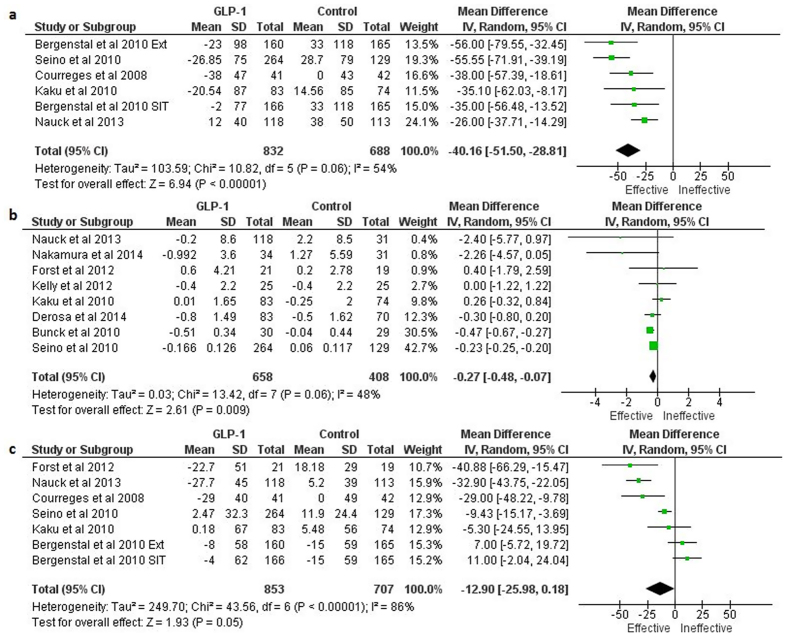
Forest plots showing the effects of GLP-1 based therapies on the percent changes from baseline of: **a**) Brain natriuretic peptide, **b**) high sensitivity c-reactive protein, and **c**) plasminogen activator inhibitor-1 levels. (In Bergenstal *et al.*, 2010: Ext, exenatide; SIT, sitagliptin).

The GLP-1 based therapies led to statistically significant reductions in the serum levels of some atherosclerosis markers. Of these, the mean difference between GLP-1 based therapies and controls in the percent changes from baseline were: −40.16 [−51.50, −28.81] %; P < 0.0001; REM (for BNP), −0.27 [−0.48, −0.07] %; P = 0.009 (for hsCRP), and −12.90 [−25.98, 0.18] %; P = 0.05; REM (for PAI-1) (Figs. 3a–c).

Decline in several atherosclerosis markers was not statistically significant after the GLP-1 based therapies. Mean differences between GLP-1-based therapy patients and controls in the changes from baseline were: IL-6 (−10.05 [−26.72, 6.63] %; P = 0.24; REM), TNF-α (−5.21 [−17.68, 7.27] %; P = 0.41; FEM), VCAM-1 (22.92 [−27.89, 73.74] ng/ml; P = 0.38; FEM) and MCP-1 (0.01 [−0.33, 0.35] μg/ml; P = 0.95; FEM). The GLP-1 based therapies were also not associated with a significant change in adiponectin levels in the blood with a mean difference in percent change from baseline between GLP-1 based therapies and controls of −53.63 [−133.86, 26.61] % (P = 0.19; REM).

The GLP-1 based therapies were also found to be associated with significant reductions in the serum levels of total cholesterol, LDL-cholesterol and triglycerides. Mean differences between GLP-1 based therapy and control patients in the changes from baseline were −5.47 [−9.55, −1.39] mg/dl (P = 0.009; REM) for total cholesterol, −3.70 [−7.39, −0.00] mg/dl (P = 0.05; REM) for LDL-cholesterol and −16.44 [−25.64, −7.23] mg/dl (P=0.0005; REM) for triglycerides (Figs S3a–c). No significant effect of GLP-1 based therapies could be noted on HDL-cholesterol (0.18 [−1.53, 1.90] mg/dl; P = 0.84; REM).

Atherosclerosis markers that were examined only in one study and therefore could not be meta-analyzed included resistin (−4.00 [−15.84, 7.84]; P = 0.51) ng/ml^16^, endothelin (0.11 [−0.03, 0.25]; P = 0.12) ng/ml^16^, pentraxin-3 (0.50 [−0.42, 1.41]; P = 0.29)^28^, c-peptide (−0.54 [−1.59, 0.52]; P = 0.32)^28^, and leptin (−1.67 [−2.64, −0.70]; P = 0.0007) μg/L^16^ (Mean differences [95% CI] in the changes from baseline between GLP-1-based therapy and control patients).

## Discussion

The GLP-1-based therapies usually utilize two strategies: 1) inhibition of dipeptidyl peptidase 4 (DPP-4) enzyme with specific inhibitors, such as sitagliptin / vildgliptin, in order to raises plasma levels of endogenous GLP-1 and GIP, and 2) utilizing DPP-4 resistant GLP-1 receptor agonists, such as liraglutide / exenatide^44^. Both these strategies are found to be successful in overcoming deficiencies of endogenous GLP-1 or providing supra-physiological levels of GLP-1.

The present study provides preliminary evidence in favor of the GLP-1 based therapies in providing beneficial effects against atherosclerosis as indicated by the significant reductions in atherosclerosis markers including BNP, PAI-1, and hsCRP, besides statistically non-significant reductions in several other markers including IMT, FMD, IL-6, and TNF-α were also noted. Moreover, GLP-1-based therapies were also associated with significant reductions in total cholesterol, LDL-cholesterol and triglycerides.

The present study finds a significant acute effect of GLP-1-based therapies in increasing % FMD but in the long term this increase was not statistically significant. Previously, it has been found that the GLP-1 infusion in healthy subjects increases baseline and acetylcholine-induced vasodilatation^45^. Upon infusion, GLP-1 exerts potent vasodilatory effects on conduit artery and arterioles in healthy humans leading to increased blood flow for enhanced microvascular recruitment and muscular perfusion^46^. This shows that GLP-1-based therapies, besides providing glycemic control, also plays a significant role in the maintenance of endothelial function and vascular health. However, for long-term effects of the GLP-1-based therapies, more trials’ data will be required.

Intima media thickness is a well-recognized marker of sub-clinical atherosclerosis^36^. Increment in carotid IMT of more than 0.34 mm/year can lead to a cardiovascular event with first myocardial infarction and stroke can happen with a carotid IMT above 0.882 mm and 0.75 mm, respectively^47^. Whereas, some longitudinal studies have noticed a significant decrease in the IMT following a GLP-1-based therapy^14^,^36^, the present meta-analysis reveals a non-significant decrease in IMT, but this may be because of the less availability of data and paradigmatic and treatment spell deviations, thus necessitates the conduct of more trials with better designs.

Blood levels of CRP above 3 mg/L are suggested to be predictive of adverse cardiovascular outcome one year later^48^. It is thought that CRP has a causative role in endothelial cell activation and dysfunction, neo-intimal formation, monocyte and macrophage activity, and matrix metalloproteinase function^49^. The present study finds that GLP-1-based therapies significantly reduce CRP while statistically non-significantly reducing TNF-α and IL-6 levels. This is suggestive of the effects of GLP-1-based therapies on attenuating inflammatory processes that may cause atherosclerosis development.

The present study finds a significant reduction in PAI-1 with GLP-1 based therapies in diabetic patients. A critical feature of atherosclerosis is the increased production of adhesion molecules from the endothelium. Deposition of cholesterol in the intima of arteries induces endothelium to produce VCAM-1^50^ which appears to be cytokine dependent as the presence of TNF-α in the smooth muscle cells is associated with an increase expression of VCAM-1^51^. In human cell culture studies, increased expressions of PAI-1 and VCAM-1 have been reported to be associated with hyperglycemia-related endothelial cell dysfunction, a process that predisposes vasculature to accelerated atherogenesis^52^.

Among the other markers of atherosclerosis, BNP, which is secreted mainly by the ventricular myocardium^53^,^54^,^55^, has been found to decrease significantly by the GLP-1-based therapies but MCP-1, which is secreted by the peripheral blood mononuclear cells^56^, has been found un-affected by the GLP-1-based therapies, in the present study. However, the present study remains inconclusive with regards to the effects on TNF-α, VCAM-1, and MCP-1, because of the unavailability of adequate data.

Taken together, results of this meta-analysis are able to provide preliminary evidence in favor of GLP-1-based therapies in overcoming atherosclerosis development/progression which is also substantiated by the significant decreases in the total cholesterol, LDL-cholesterol and triglycerides observed in the studies which fulfilled inclusion criteria of this study.

Of the limitations of the present study, owing to the availability of fewer studies in some comparisons of individual parameters, it remains under-powered to generate conclusive evidence. Methodologically, among the included longitudinal studies, only 9 were double blind RCTs, 11 were open-label RCTs and 4 were non-randomized observational studies. This may also affects the level of evidence and thus the outcomes of this analytical review should be assigned as preliminary evidence only. Funnel plots also reflected a considerable publication bias (Fig. S2). In some comparisons, statistical heterogeneity was high; although, sensitivity analyses revealed only one comparison with truly higher I^2^. For this reason, random effects model has been used to interpret the results of the comparisons with high heterogeneity, yet, future randomized studies with better designs will be required to further assess these outcomes.

## Conclusion

This study provides preliminary evidence in support of GLP-1 based therapies in providing beneficial effects against atherosclerosis as this therapeutic regimen was found to be associated with statistically significant reductions in the plasma levels of atherosclerosis markers including plasminogen activator inhibitor-1, high sensitivity c-reactive protein, and brain natriuretic peptide. Those makers which are found to be statistically non-significantly reduced by the GLP-1-based therapies include tumor necrosis factor-α and interlekin-6. This study further strengthens the existing evidence in favor of GLP-1 based therapies in decreasing total cholesterol, LDL-cholesterol and triglycerides. More randomized controlled and blinded trials will be required to arrive at conclusive evidence.

## Additional Information

**How to cite this article**: Song, X. *et al.* Anti-atherosclerotic effects of the glucagon-like peptide-1 (GLP-1) based therapies in patients with type 2 Diabetes Mellitus: A meta-analysis. *Sci. Rep.* **5**, 10202; doi: 10.1038/srep10202 (2015).

## Supplementary Material

Supplementary figures

## Figures and Tables

**Table 1:** Summary of the met-analyses assessing the effects of GLP-1-based therapies on various markers of atherosclerosis.

Parameter	No. of studies	No. of subjects	Mean difference [95% CI]; Significance level; Model	I^2^	Remarks
FMD (%)					
Change from baseline	6	278	0.83 [−1.06, 2.73]; P = 0.39; REM	90%	GBT non-significantly increased % FMD
GBT vs control	3	99	1.65 [−0.89, 4.18]; P = 0.2; REM	76%	GBT non-significantly increased % FMD
Cross sectional studies	7	198	2.58 [1.68, 3.53]; P < 0.00001; REM	96%	GBT significantly increased % FMD
cIMT (mm)					
Change from baseline	6	424	−0.06 [−0.15, 0.03]; P = 0.18; REM	61%	GBT non-significantly decreased cIMT
GBT vs control	2	166	−0.04 [−0.09, 0.01]; P = 0.1; FEM/REM	0%	GBT non-significantly decreased cIMT
Adiponectin (% change)	6	957	−53.63 [−133.86, 26.61]; P = 0.19; REM	99%	Indifferent
TNF-α (% change)	2	236	−5.21 [−17.68, 7.27]; P = 0.41; FEM/REM	0%	Indifferent
IL-6 (% change)	4	317	−10.05 [−26.72, 6.63]; P = 0.24; REM	41%	Indifferent
hs-CRP (mg/l)	8	1066	−0.27 [−0.48, −0.07]; P = 0.009; REM	48%	GBT non-significantly decreased hs-CRP
PAI-1 (% change)	7	1507	−12.90 [−25.98, 0.18]; P = 0.05; REM	86%	GBT significantly decreased PAI-1
BNP (% change)	6	1520	−40.16 [−51.50, −28.81]; P<0.0001; REM	54%	GBT significantly decreased BNP
MCP-1 (% change)	2	99	0.01 [−0.33, 0.35]; P = 0.95; FEM	0%	Indifferent
VCAM-1 (ng/ml)	2	90	22.92 [−27.89, 73.74]; P = 0.38; FEM	0%	Indifferent
Total cholesterol (mg/dl)	7	1048	−5.47 [−9.55, −1.39]; P = 0.009; FEM	24%	GBT significantly decreased cholesterol
HDL-cholesterol (mg/dl)	9	1092	0.18 [−1.53, 1.90]; P = 0.84; REM	60%	Indifferent
LDL-cholesterol (mg/dl)	7	1007	−3.70 [−7.39, −0.00]; P = 0.05; REM	65%	GBT significantly decreased LDL-chol.
Triglyceride (mg/dl)	10	1122	−16.44 [−25.64, −7.23]; P = 0.0005; REM	46%	GBT significantly decreased LDL-chol.

Abbreviations: cIMT, carotid intima media thickness; FEM, fixed effects model; FMD, flow-mediated diameter; GBT, GLP-1-based therapies; HDL, high-density lipoprotein; hs-CRP, high sensitivity C-reactive protein; IL-6, interleukin-6; LDL, low-density lipoprotein; MCP-1, monocyte chemotactic protein-1; PAI-1, plasminogen activator inhibitor-1; REM, random effects model ; TNF-α, tumor necrosis factor-α; VCAM-1, vascular cell adhesion molecule-1.
