# Supplementary material for: Anti-atherosclerotic effects of the glucagon-like peptide-1 (GLP-1) based therapies in patients with type 2 Diabetes Mellitus: A meta-analysis
Source: Sci Rep. 2015 Jun 26;5:10202. doi: 10.1038/srep10202 (PMC4481643; doi:10.1038/srep10202)
Supplement: Supplementary figures [file srep10202-s1.doc]

Original Article

**Anti-atherosclerotic effects of the glucagon-like peptide-1 (GLP-1) based therapies in patients with type 2 Diabetes Mellitus: A meta-analysis**

Xiaoyan Song^1,2^, Hetang Jia^2^, Liang Wang^3^, Yan Zhang^3^, Yiming Mu^1,*^, Yu Liu^4,*^

^1^Department of Endocrinology, Chinese PLA General Hospital, Beijing 100853, China;

^2^Department of Endocrinology, Chinese PLA 309 Hospital, Beijing 100091, China;

^3^Department of Orthopedics, Chinese PLA 309 Hospital, Beijing 100091, China;

^4^Department of Geriatric Endocrinology, General Hospital of PLA, Beijing 100853, China

***Co-corresponding author:**

**Dr. Yu Liu**

Department of Geriatric Endocrinology, General Hospital of PLA, No.28 Fuxing Road, Beijing 100853, China

Tel: +86-10-55499001 Fax: +86-10-55499001

Email: Liuyu1227@hotmail.com

**Dr. Yiming Mu**

Department of Endocrinology, Chinese PLA General Hospital, No.28 Fuxing Road, Beijing 100853, China

Tel: +86-10-55499001 Fax: +86-10-55499001

Email: [muyiming@301hospital.com.cn](mailto:muyiming@301hospital.com.cn)

**Running title**: Anti-atherosclerotic effects of the GLP-1 based therapies


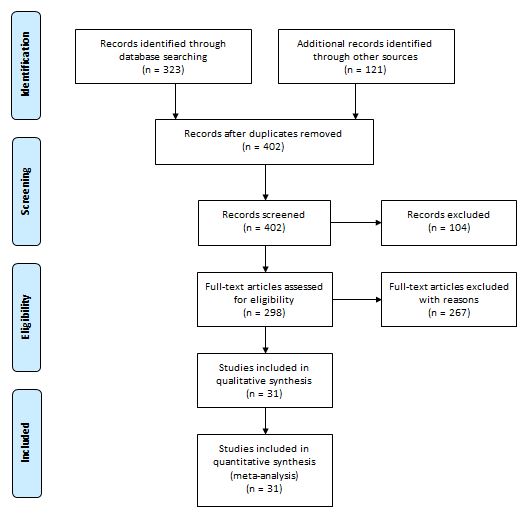


Figure S1: A flowchart of study screening and selection process


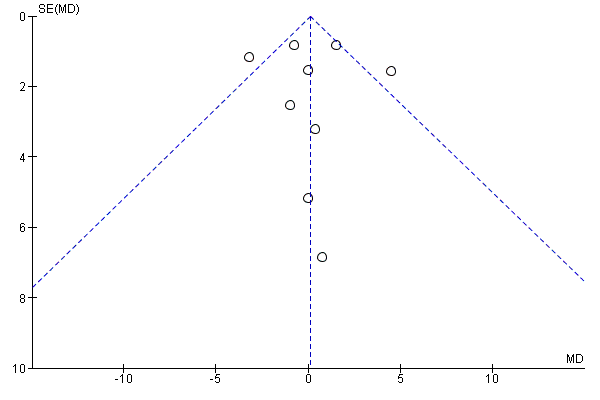


Figure S2: Funnel plot of a meta-analysis (effect of GLP-1-based therapies on LDL-cholesterol) showing publication bias

| a | 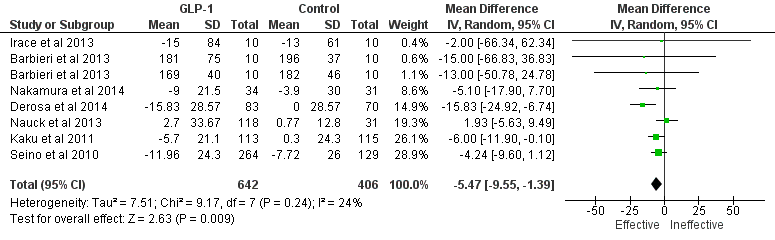 |
| --- | --- |
| b | 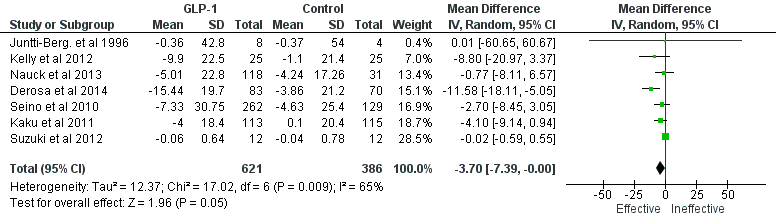 |
| c | 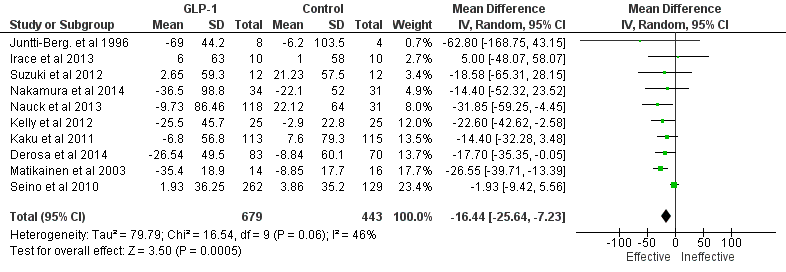 |

Figure S3: Forest plot showing the effects of GLP-1 based therapies on: a) total cholesterol, b) LDL-cholesterol, and c) triglycerides.
